# Speaking Up and Speaking Out: Gender Diversity in the Scientific Programme of the Royal Australasian College of Surgeons Annual Scientific Congress

**DOI:** 10.1007/s00268-021-06105-4

**Published:** 2021-07-17

**Authors:** Christine S. Lai, Jessica Farrar, Fellicia Stanzah, Bradley Crammond, Sandra L. Wong, James C. Lee

**Affiliations:** 1 Division of Surgery, The University of Adelaide, Adelaide, SA Australia; 2 Department of Surgery, The Queen Elizabeth Hospital, Central Adelaide Local Health Network, Adelaide, SA Australia; 3 School of Population and Global Health, University of Melbourne, Carlton, VIC Australia; 4 Department of Surgery, Dartmouth-Hitchcock Medical Center, Lebanon, NH USA; 5 The Dartmouth Institute for Health Policy and Clinical Practice, Lebanon, NH USA; 6 Department of Surgery, Central Clinical School, Monash University, Melbourne, VIC Australia; 7 Department of General Surgery, The Alfred Hospital, Melbourne, VIC Australia

## Abstract

**Background:**

Disparities in gender representation at medical meetings have been documented despite women representing half of medical school graduating classes. Lack of role models is touted as one of a myriad of factors that perpetuate gender imbalance, particularly in the field of surgery. We evaluated the trend in gender distribution of participants at the Royal Australasian College of Surgeons (RACS) Annual Scientific Congress (ASC) and whether there was a correlation between the gender distribution of the organising committee and speakers and chairpersons invited to attend.

**Methods:**

RACS ASC programmes from 2013 to 2018 were retrospectively analysed, examining the gender distribution of speakers, chairpersons and conveners. Trend analysis of distribution was performed, and a generalized linear mixed model was used to investigate the effect of the gender of the conveners on gender of session chairpersons and speakers.

**Results:**

Between 2013 and 2018, there were non-significant increases in female speakers invited to speak from 14.9 to 21.7% (*p* = 0.064) and female conveners appointed from 11 to 19% (*p* = 0.115), but there was a significant increase in female chairs from 9.6 to 21.6% *p* < 0.001). Female conveners were 3 times more likely to invite female speakers than male conveners (*p* < 0.001) and were 20 times more likely to invite female chairs than male conveners (*p* < 0.001).

**Conclusion:**

Visible role models are important in the pursuit of gender equity in surgery in order to break down stereotypes and the hidden curriculum. Intentional effort is required to achieve parity, and such efforts could include appointing more women to organising committees of scientific meetings.

## Introduction

A myriad of factors influence a young medical professional in their career choice. The interplay of these factors is complex and poorly understood. It has been reported that some of these factors are barriers which may deter women from choosing a career in surgery [1–3]. In many countries, more than 50% of medical school graduates are women [4, 5]; however, disparities still exist in the proportion of women choosing to pursue a career in surgery. In 2018 in Australia and New Zealand, women represented 30% of applicants for the Royal Australasian College of Surgeons (RACS) surgical training programmes, 29% of successful applicants, and 27% of the new fellows [6].

Academic meetings not only provide educational opportunities for students and junior doctors, but also to network with and be inspired by senior colleagues. Unfortunately, in many large medical and surgical conferences, the proportion of female speakers is far exceeded by their male counterparts [3, 7–13]. This kind of underrepresentation of women in can negatively impact on the future of aspiring junior doctors due to the lack of visible role models [2, 3, 14].

The RACS Annual Scientific Congress (ASC) is the largest, cross-disciplinary surgical meeting held in Australia and New Zealand and is the flagship professional development event of the college. In addition to providing medical education, the ASC functions as an important platform for professional networking, establishing formal and informal mentorships, and a place where role models can be seen and heard.

Recognising the importance of diversity and inclusion in the surgical workforce, the RACS published its “Diversity and Inclusion Plan” in late 2016 [15]. The plan was developed from the evidence that having a diverse surgical workforce is associated with improvements in patient outcomes. It sets an organisation-wide, aspirational target of 40% women in leadership positions, committee roles and annual trainee intake by 2021. The plan also included strategies to improve the visibility of female surgeons in print and social media.

We set out to determine the trend in the visibility of women role models within the RACS ASC by (1) evaluating the trend in gender distribution of speakers and chairpersons; (2) determining the proportion of women in the RACS ASC organising committees; and (3) determining if a correlation exists between the gender distribution of organising committee members and podium appearances at the RACS ASC.

## Methodology

For each ASC, an overall *Congress Convener,* a *Scientific Convener* and executive committee are appointed to steer the conference and organise plenary sessions. The RACS regional committee hosting the meeting appoints those positions. *Section Conveners* are appointed by the surgical sections committees to convene surgical subspecialty or cross-disciplinary programmes within the conference. They are responsible for the programming of their section and extending invitations to the *Invited Speakers, ASC visitors* (pre-eminent *Invited Speakers* funded by RACS) and *Chairpersons. Podium appearances* consist of both *Invited Speakers* and *Chairpersons*.

The ASC program database was obtained for meetings between 2013 and 2018 inclusive. A review was undertaken of the profiles and roles of all organising committee members, speakers and chairpersons. The gender of each individual was determined using a combination of their biography, Australian Health Practitioners Regulation Agency, registry of health practitioners and Medical Council of New Zealand, as well as online search results.

To investigate the effect of the *Section Convener* gender on the gender of speakers, we used a generalised linear mixed model. The primary outcome was a dichotomous gender variable for speaker (male/female) and a random intercept was designated for the primary covariate of interest, an unordered three-level indicator of *Section Convener* gender (male/female/mixed). In the case of an individual *Section Convener*, gender was recorded according to that individual’s gender. In the case of multiple conveners for one section, gender was male/female when all *co-conveners* were of that gender and was coded ‘mixed’ when there were both males and females making up the *co-convener* panel. A mixed model was used to account for within *Section Convener* correlation between speaker choices, that is, to account for some Section Conveners being individually more or less likely to choose speakers of a certain gender.

All results have been adjusted for the year of the conference, treated as a continuous variable, and whether or not the speaker was an ASC visitor. We did not adjust for whether or not a speaker gave multiple talks owing to collinearity with invitation. Statistical analysis was performed using Stata 16 using significance levels of *p* < 0.05 (StataCorp LLC, College Station, TX).

## Results

### Gender distribution of invited speakers and chairpersons

During the 6 year study period, there were a total of 6792 *podium appearances*. Of these, 6709 were included for analysis after excluding 84 with missing data. There were 4713 *Invited Speakers* (912 female, 3801 male), of which there were 288 *ASC visitors* (45 female, 243 male) and 1965 *Chairpersons* (303 female, 1662 male). Over the 6 years, once adjusted for the effect of *Section Convener* gender, there was a non-significant increase in female *Invited Speakers* of 8.7% (95% CI 0.5–18%, *p* = 0.064) but a significant increase in female *Chairpersons* of 26% (95% CI 7–47% *p* = 0004).

The number of female *ASC visitors* varied from as low as 4% in 2016 to 18% in 2018, with a mean of 16%. Women were 30% less likely to be invited as an *ASC visitor* than men (95% CI: 10–50%, *p* < 0.004) (Table 1). *ASC visitors* were 81 times more likely to give multiple presentations than an *Invited Speaker*.

**Table 1:** Gender breakdown of invited speakers, chairpersons, ASC visitors and composition of ASC organising committees from 2013 to 2018

Year	2013	2014	2015	2016	2017	2018	Totals	*P* value
*Invited speakers*								
N	619	880	682	837	675	1024	4717	
F:M	92:527	142:738	144:538	176:661	135:540	223:801	912:3801	
% F	14.9	16.1	21.1	21.0	20.0	21.8	19.3	0.064
*Chair persons*								
N	269	327	295	332	319	425	1967	
F:M	26:243	42:285	45:250	52:280	46:273	93:332	303:1662	
% F	9.7	12.8	15.3	15.7	14.4	21.9	15.4	0.0004
*ASC visitors*								
N	39	56	41	45	45	62	288	
F:M	6:33	9:47	9:32	2:43	10:35	9:53	45:243	
%F	15.4	16.1	22.0	4.4	22.2	17.7	16.3	0.844
*Congress convener*								
Gender	M	M	M	M	M	M	6 M	
*Scientific convener*								
Gender	M	M	F	M	M	F	2F, 4 M	
*Executive committee members*								
N	4	4	3	4	3	3	21	
F:M	1:3	1:3	0:3	0:4	1:2	0:3	3:18	
%F	25	25	0	0	33	0	14.2	
*Section convener*								
N	27	42	32	46	36	47	230	
F:M	3:24	10:32	3:29	8:38	7:29	9:38	40:190	
%F	11	24	9	16	19	19	17.3	0.115
*Total organising committee members*								
N	33	48	37	52	41	52	263	
F:M	4:29	11:37	4:33	8:44	8:33	10:42	45:218	
%F	12	23	11	15	20	19	17.1	0.636

**Fig. 1:**
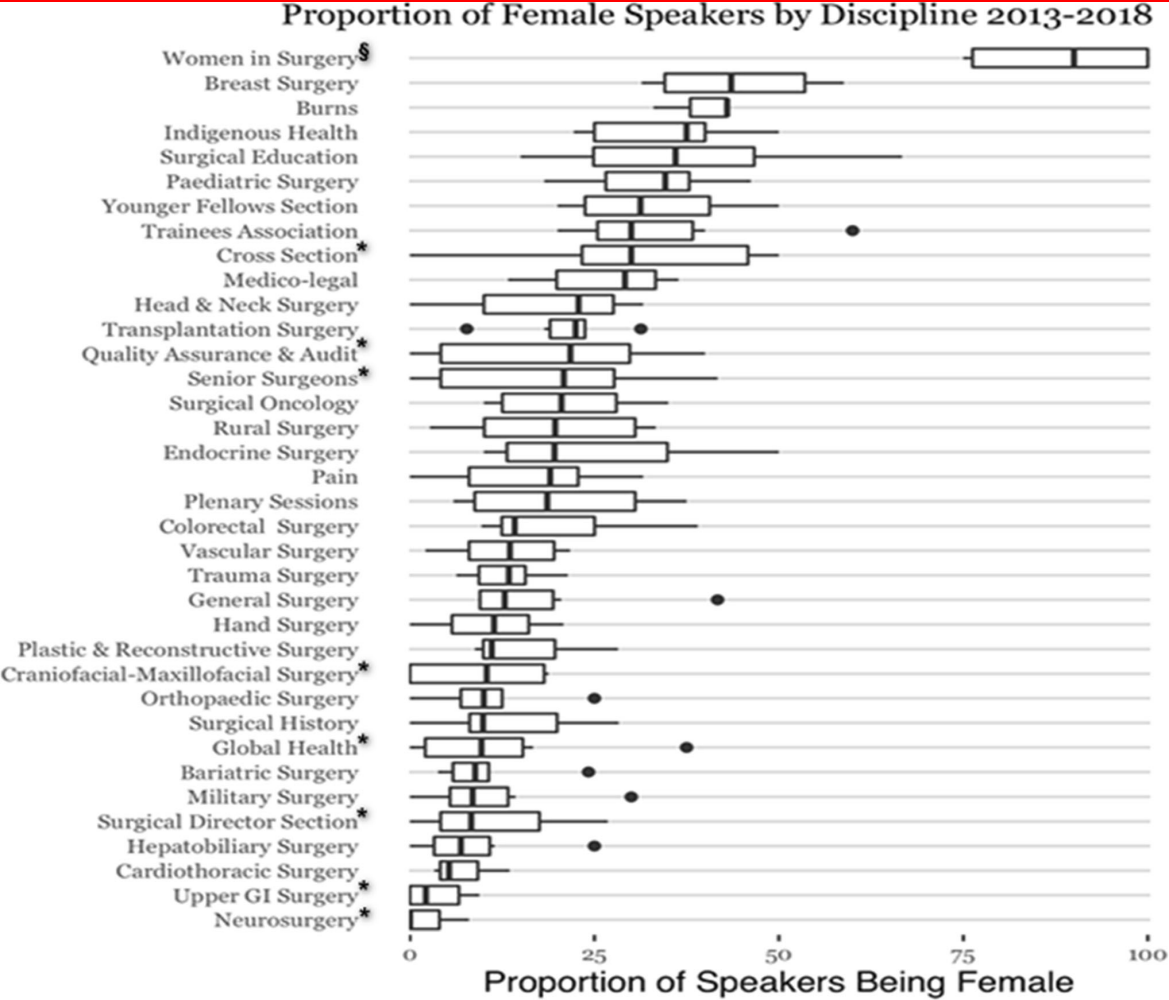
Whisker and box plot demonstrating proportion of female invited in the RACS ASC by Sects. 2013–2018. * multiple years of male only invited speakers, § multiple years of female only speakers

In this period, there were 36 sections convened within the ASC programme. Of these, 20 sections were scientific streams of recognised surgical specialties or subspecialties, and 16 were cross-disciplinary sections of interest to all surgeons, including plenaries. There were seven scientific streams and eight cross-disciplinary streams with a programme featuring male *Invited Speakers* only in at least one of the 6 years, with cross section, quality assurance and audit, senior surgeons, craniofacial-maxillofacial surgery, global health, surgical director, upper gastrointestinal and neurosurgery sections having male only speakers in 2 or more years (Fig. 1).

A logistic regression of speaker gender on proportion of females in the specialty showed that a 1% increase in female fellows was associated with a 5% increase in female speakers adjusting for effect of female conveners (95% CI 1.01–1.09, *p* value = 0.007).

Women in surgery had the highest female *Invited Speaker* percentages ranging from 75 to 100% (mean 85.7%), with female-only programmes in three of the years. The next highest sections with female speaker representations were breast surgery with 43.9% (range 31.4%–58.8%) and paediatric surgery with 31.7% (range 18.2–46.2%) (Fig. 1). The Women in surgery and breast surgery sections were outliers with a disproportionately higher number of women speakers in their programmes compared to the proportion of female surgeons within the specialty or section. Conversely, the streams with the lowest percentage of female *Invited Speakers* were neurosurgery (mean 2.9%), upper gastrointestinal (mean 4.3%), surgical directors (mean 6.3%) and cardiothoracic surgery (mean 7.2%) (Table 2).

**Table 2:** Average percentage of female invited speakers in each specialty and cross-sectional interest sections from 2013 to 2018 compared to the average percentage of female surgeons in the specialty or in Australia and New Zealand. Quartile ranking is also noted

Specialty section	Ave female speakers %	Ave female in specialty %
*1st Quartile*		
Neurosurgery	2.9	11.8
Upper GI surgery^1^	4.3	14.7^1^
Cardiothoracic surgery	7.2	6.7
Hepatobiliary surgery^1^	7.7	14.7^1^
Bariatric surgery^1^	10.6	14.7^1^
*2nd Quartile*		
Trauma surgery^2^	12	10.8^2^
Craniofacial-maxillofacial surgery^3^	12.1	13.8^3^
Orthopaedic surgery	12.4	3.9
Plastic and reconstructive surgery	13.9	13.8
Vascular surgery	13.9	9.9
*3rd Quartile*		
Hand surgery	14.4	3.9
General surgery	15.8	14.7
Colorectal surgery^1^	19.8	14.7^1^
Head and neck surgery	20	13.4
Surgical oncology^2^	20.1	10.8^2^
*4th Quartile*		
Transplantation surgery^1^	21.7	14.7^1^
Endocrine surgery^1^	26.0	14.7^1^
Paediatric surgery	31.7	26.5
Burns^3^	37.8	13.8^3^
Breast surgery^1^	43.9	14.7^1^

Cross-sectional sections	Ave female speakers%	Ave female surgeons %^2^
*1st Quartile*		
Surgical director section	6.3	10.8
Military surgery	12.3	10.8
Global health	13.7	10.8
Surgical history	14.4	10.8
*2nd Quartile*		
Pain	18.5	10.8
Rural surgery	18.5	10.8
Plenary sessions	22.4	10.8
Senior surgeons	23.3	10.8
*3rd Quartile*		
Quality assurance and audit	25.2	10.8
Medico-legal	25.6	10.8
Cross section	27.0	10.8
Younger fellows section	30.0	10.8
*4th Quartile*		
Trainees association	32.5	10.8
Indigenous health	33.3	10.8
Surgical education	34.2	10.8
Women in surgery	85.7	10.8

Gender proportion data are unavailable for the specialty or section, percentages of female surgeons in ^1^General surgery, ^2^Entire RACS fellowship and ^3^Plastic surgery are listed

### Gender distribution of ASC organising committee members

There were no female *Congress Conveners* and two female *Scientific Conveners* throughout the study period. Female representation remained low in the executive committees. The proportion of female *Section Conveners* increased from 11.1 to 19.1% from 2013 to 2018, though this was not a monotonic trend (Table 1).

### Association of section convener gender and gender of invited speakers/chairpersons

Adjusting for the 6 year trend and whether or not the *Invited speaker* was an *ASC visitor*, female *Section Conveners* were over three times more likely to include female *Invited Speakers* in the programme than male *Section Conveners* (odds ratio 3.17, 95% CI 2.01–5.00, *p* value < 0.001). Mixed-gender *Section Co-convener* pairs were 1.6 times more likely to invite a female *Invited Speakers* than a male *Section Convener* alone (95% CI: 0.92–2.71, *p* = 0.094).

Adjusting for the 6 year trend, female *Section Conveners* were nearly 20 times more likely to appoint female *Chairpersons* than male *Section Conveners* (95% CI 8.66–44.86, *p* value < 0.001). Mixed-gender *Section Co-convener* pairs were five times more likely to appoint female *Chairpersons* than male *Section Conveners* (95% CI 2.09–12.26, *p* value < 0.001).

## Discussion

Our study shows that overall representation of female *Invited Speakers* at the RACS ASC was 19.3% over 6 years, which showed a statistically non-significant increase from 14.9 to 21.7% between 2013 and 2018. These figures compare favourably with the proportion of female surgeons in Australia and New Zealand which increased from 9.8 to 12.1% during the same timeframe [6]. The proportion of women *Section Conveners* increased from 11 to 19% over this time period, which correlated strongly to the increase in female *Invited Speakers.* We also found that the composition of ASC committees had a lower representation of women (17%) than the aspirational target of 40% set by RACS in 2016. No woman was appointed as the *Congress Convener* and a woman was appointed as *Scientific Convener* in two of the 6 years.

One of the key findings of this study is that a female *Section Convener* invited more than three times the proportion of female *Invited Speakers*, when compared with a male *Section Convener*. Even the pairing of female and male *Section Co-conveners* was associated with increase in female *Invited Speakers*, albeit non-significantly, compared to a male *Section Convener*. This concurs with the findings of other studies which have shown that women on organising committees significantly increasing the proportion of female podium presenters in the fields of science [3, 14, 16–20]. Several studies have also reported the underrepresentation of female conference speakers in the fields of critical care, obstetrics and gynaecology, anaesthesia, emergency medicine, and internal medicine, relative to the gender distribution of their specialty memberships and/or conference registrants [8–11]. However, in those studies, the gender makeup of the conveners was not specifically examined for their direct impact on the gender of the speakers invited to speak.

It has been proposed that *Section Conveners* are likely subject to affinity biases for the same gender and other attributes, in addition to other implicit biases. Therefore, for a male *Section Convener*, the effects of these biases can compound to result in an unconscious and unintentional preference for male invitees over women [21]. It has also been reported that women may be under-sponsored and less frequently proposed as speakers, possibly due to implicit biases [22]. For these reasons, genuine attempts to increase gender diversity within the conference programme must include appointing a reasonable proportion of woman in organisational roles, and take invitations both to chair and to speak in a session into account.

The issue of defining gender balance of conference speakers has increasingly been a topic of discussion in academic publications and social media [12, 19, 20, 23]. Adjusting for the effect of *Section* C*onvener* gender, for every 1% increase in female surgeons in a specialty, there was a 5% increase in female speakers. This suggests that as the pool of speakers increases, it should become incrementally easier to find female speakers. Our data are at odds with the notion that gender imbalance is due to women declining invitations to speak [9, 12, 18, 19]. The findings in this study are similar to figures published on North American surgical conferences, where the mean proportion of female speakers exceeds the proportion of women in the surgical workforce. Ruzycki et al. reported that between 2007 and 2017, the proportion of female surgeons in North America increased from 14.8 to 18.3%, while the proportion of female speakers in surgical conferences increased from 20.1 to 28.4% [13].

In this study, the women in surgery section was a notable outlier where the proportion of female speakers (mean 83%) far exceeded the proportion of female surgeons in the cross-disciplinary streams (mean 10.8% female surgeons). This is likely due to the topics chosen for discussion relating specifically to women. Most cross-disciplinary streams also had female representation exceeding the proportion of female fellows, except Surgical Directors.

Of the specialty streams, the highest average of 44% women *Invited Speakers* was the breast surgery section, a subspecialty of general surgery. The percentage of female surgeons in the field of breast surgery is higher than in general surgery (14.7%) [24]. On the opposite end of the spectrum, neurosurgery, upper gastrointestinal, cardiothoracic, hepatobiliary and bariatric surgery were in the lowest quartile of female representation. These sections had nearly exclusively male *Section Conveners* during the study period, which likely had contributed to the low percentages of female *Invited Speakers*.

Approximately 50% of medical school graduates, 29% of surgical trainees, 27% of younger fellows and 12% of surgeons are women in Australia and New Zealand [4–6]. These figures represent both changing demographics of surgeons as well as a “leaky pipeline” of women choosing a career other than surgery. Therefore, it is essential that women surgeons are visible on the podium in conferences to help soften gender stereotypes and provide role models for women aspiring to become surgeons [2, 12].

The *RACS Diversity and Inclusion Plan* came into effect as the 2018 ASC was being organised [16]. While trending in the right direction, our results show that the proportion of women on the organising committee and women invited to speak at the ASC have some way to go to reach the target of 40%. This target has been touted as representative of societal gender balance [21]. A gender-balanced conference programme is possible to achieve with intentional effort by organisers even in fields which have low proportion of female specialists. For example, the social media and critical care conference advocates all forms of diversity and always intentionally achieves gender parity of speakers [25]. In order to achieve set targets, in addition to increasing the proportion of women in organisational roles, new strategies including development of speaker policies [12, 17] building and maintaining databases of women speakers may need to be considered at an organisational level.

Limitations of our study include possible attribution errors since publicly available databases were utilised to determine genders of individuals and assumptions regarding biological sex were used rather than the gender the person may identify as. We were also unable to account for other aspects of diversity such as race/ethnicity, religion, sexual orientation, age and academic seniority, and therefore unable to assess the impact of intersectionality on inclusion in speaking roles at conferences.

## Conclusion

Our study confirms that a higher proportion of women speakers and chairpersons are invited when a female *Section Convener* is appointed to convene a specialty stream within the ASC. The increase in invited female speakers over time was likely a result of the increase in women appointed to the organising committee. It is important to achieve or exceed the RACS Diversity and Inclusion target of 40% women when forming future ASC organising committees, as gender-balanced organising committees will continue to improve on the numbers of invited female speakers. Intentional efforts will still be required in order to achieve gender equity of speakers at conferences in order to provide visible role models to students and junior doctors considering a career in surgery.
